# Increased Rider Weight Did Not Induce Changes in Behavior and Physiological Parameters in Horses

**DOI:** 10.3390/ani10010095

**Published:** 2020-01-06

**Authors:** Janne Winther Christensen, Suzie Bathellier, Marie Rhodin, Rupert Palme, Mette Uldahl

**Affiliations:** 1 Department of Animal Science, Aarhus University, Blichers Allé 20, 8830 Tjele, Denmark; 2 Agrocampus Ouest, 65 Rue de Saint-Brieuc, 35042 Rennes, France; suzie.bathellier@agrocampus-ouest.fr; 3 Department of Anatomy, Physiology and Biochemistry, Swedish University of Agricultural Sciences, Box 7011, 75007 Uppsala, Sweden; Marie.Rhodin@slu.se; 4 Department of Biomedical Sciences, University of Veterinary Medicine, Veterinärplatz 1, A-1210 Vienna, Austria; rupert.palme@vetmeduni.ac.at; 5 Vejle Hestepraksis, Fasanvej 12, 7120 Vejle Oest, Denmark; mette@vejlehestepraksis.dk

**Keywords:** behavior, dressage horse, gait symmetry, rider weight, saliva cortisol, welfare

## Abstract

**Simple Summary:**

The influence of rider weight on horse welfare, health and performance is often debated. We measured the effects of increasing the weight of the regular rider by 15% and 25% on horse behavior, gait symmetry and physiological responses in a standard dressage test. Cortisol levels increased in response to exercise, but we found no effect of the weight treatment, i.e., cortisol levels did not increase when the rider became heavier. Behavior, heart rate and gait symmetry also did not differ between treatments. We conclude that increasing the weight of the regular rider by 15% and 25% did not result in significant short-term alterations in cortisol, heart rate, behavior and gait symmetry in horses during low-intensity exercise. Further studies are required to develop appropriate guidelines for rider weight.

**Abstract:**

Recent studies have reported significant alterations in horse physiological and gait parameters when exposed to increased rider weight during moderate to high intensity exercise. This study aimed to evaluate the effect of increased rider weight (+15% and +25% of the regular rider’s bodyweight) on horse behavioral, physiological and gait symmetry parameters during a standard dressage test. Twenty rider-horse equipages performed the same test three times in a randomized, crossover design. Salivary cortisol (SC), heart rate (HR), heart rate variability (HRV), behavior and gait symmetry (GS) were measured. SC concentrations increased from baseline (*p* < 0.001), but there was no significant treatment effect (difference from baseline (ng/mL): Control: 0.21 ± 0.1; +15%: 0.37 ± 0.1; +25%: 0.45 ± 0.2, *p* = 0.52). Similarly, there were no overall treatment effects on HR or HRV variables (avg HR across treatments (bpm): 105.3 ± 1.3), nor on GS parameters. There was large individual variation in conflict behavior but no effect of weight treatment. We conclude that increasing the weight of the regular rider by 15% and 25% did not result in significant short-term alterations in the measured parameters. Maximum rider:horse weight ratios were 15–23% and the exercise intensity was relatively low; thus the results should not be extrapolated to other weight ratios and exercise intensities.

## 1. Introduction

The impact of rider weight on horse welfare, health and performance is frequently debated, especially as the human population gets heavier [1]. Previous studies have reported that horse behavioral, physiological and gait parameters can be negatively affected by increased rider weight [2,3,4,5]. However, the effects on horse welfare are likely influenced by many factors, such as horse fitness and muscle development, conformation, discipline (e.g., show jumping or dressage), exercise intensity, the rider’s fitness, skill and balance as well as equipment used, e.g., saddle fit [5]. Thus, multiple studies focusing on these aspects and targeting the issue of rider weight from different perspectives are needed before appropriate guidelines can be developed. The subject is relatively under-studied in warmblood horses as most studies have used other breeds, e.g., Icelandic horses, which are commonly subjected to higher rider:horse bodyweight ratios than that common for larger warmblood horses; and/or used exercise intensities that are higher than that to which many ordinary riding horses are usually exposed. In two of these studies on Icelandic horses, Stefánsdóttir et al. [2] and Gunnarsson et al. [3] explored the effects of increasing the rider:horse bodyweight ratio within the commonly experienced range by Icelandic horses (20% to 35%) on physiological and gait (tölt) parameters, during high intensity exercise. One rider rode eight horses in an incremental test, where the weight ratio increased for each phase. Average heart rates increased significantly from the 20% (187 bpm) to the 35% phase (199 bpm). Similarly, plasma lactate concentrations, rectal temperature and breathing frequencies increased with increasing weight ratio [2]. In addition, the weight increment decreased stride length whereas parameters such as beat, symmetry and height of front leg lift appeared unaffected [3]. Gait analysis was also used in a series of studies on native Japanese horses and ponies by Matsuura et al. [6,7,8,9]. The horses/ponies trotted a short (40 m) straight distance with weights up to 130 kg, and symmetry, regularity and stability of the locomotion rhythm were recorded. The authors reported that gait symmetry parameters were interrupted when the horses were carrying 29–43% of their bodyweight during this short, low-intensity exercise. Thus, small horses and ponies can be negatively affected by high rider:horse bodyweight ratios at both high and low exercise intensities.

Three studies investigated effects of rider weight in warmblood horses. One of these studies was a treadmill study [10], where nine warmblood horses were exercised unmounted and mounted with a 90 kg rider or 90 kg of dead lead weight (mount:horse bodyweight ratios: 13–16%). Peak heart rates and plasma lactate concentrations were significantly lower in unloaded horses, with no differences between mounted and lead-loaded horses. Stride parameters were also affected in that, e.g., the relative stance duration in trot increased when mounted and loaded [10]. In another study, Powell et al. [4] exposed eight horses—described as light type riding horses—to a riding test of a relatively high intensity where they carried 15%, 20%, 25% and 30% of their own bodyweight in a crossover study. The horses were ridden by one of three experienced riders, and additional weight was attached to the saddle. The horses had increased heart rates, respiration rates and rectal temperatures post-exercise in the 25% and 30% treatment, as well as increased lactate concentrations and muscle soreness after the 30% treatment [4]. Finally, in a recent pilot study, Dyson et al. [5] exposed six warmblood horses to four riders of different weights (61–142 kg). The riders were classified as light to very heavy based on the rider:horse bodyweight ratios (Light: 10–12%; Moderate: 13–15%; Heavy: 15–18%; Very heavy: 24–28%). Based on a subjective scoring of lameness and behavioral markers of pain, all riding tests with the heavy and very heavy riders were abandoned on welfare grounds. However, since four different riders comprised the treatment, the results may relate to differences in riding style or level, rather than increased rider weight per se. It should also be noted that the previous studies have included a limited number of horses (n = 6–10 horses). Thus, although some evidence exists for negative effects of increased rider weight at high rider:horse bodyweight ratios (25–30%) at relatively high exercise intensities, it is relevant to gain more knowledge of how rider weight influences horse welfare at ratios and exercise intensities typical for warmblood leisure horses. In Scandinavia, the average bodyweight of adult women is 67–73 kg and men: 82–88 kg [2]. Consequently, for warmblood horses with a bodyweight of 500–600 kg and with a tack weight of 10–15 kg, rider:horse bodyweight ratios of 14–18% are common. 

The aim of the present study was to investigate the effect of acute increases in rider weight on behavioral, physiological and gait parameters in horses during a standard, low-intensity riding situation. We used a randomized and balanced crossover design, where 20 riders rode their own horse in a standard dressage test with either no added weight, extra 15% or extra 25% of their bodyweight. Thus, we were able to isolate the effect of increased rider weight while keeping other factors identical. We hypothesized that added weight would lead to increased salivary cortisol and heart rate responses, more conflict behavior and increased gait asymmetry.

## 2. Materials and Methods

Twenty female riders and their horses (6 mares, 12 geldings and 2 stallions) were included in the study (Table 1). All horses were active riding and competition horses. We initially aimed to include an equal number of horses and ponies but due to safety issues with attaching lead weight to the upper body of children, we had to cut down on the number of participating ponies. The initial average rider:horse bodyweight ratio was 15.3% ± 0.4 (range 12–19%); with added 15%: 17.2% ± 0.5 (range 14–21%); and with added 25%: 18.5% ± 0.5 (range 15–23%). Rider weight included tack for the calculation of rider:horse bodyweight ratios.

**Figure 1:**
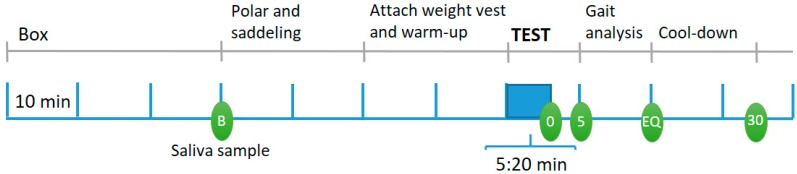
Daily procedure for the riding tests on Test Days 2–4. Five saliva samples were collected from each horse per day: B: Baseline; 0: Just after the dressage test; 5: 5 min after dressage test, EQ: Just after the saddle pressure/gait symmetry test, 30: 30 min after the dressage test.

The 20 equipages were tested in two blocks of 10, with each block lasting four days (two successive weekends). All days followed the procedure illustrated in Figure 1, except the first day, where baseline data were collected and the horses were habituated to the experimental set-up and equipment. On this first day, a standard (pre-purchase) clinical examination was performed by an experienced equine vet after collection of the baseline saliva sample, ensuring that the horses were sound for the experiment. Heart rate monitoring equipment (see Section 2.1) was then fitted and the horse performed a baseline lunge test with the same amount of physical exercise as the riding test on Days 2–4 (5:20 min: 10 s walk, 1 min trot, 1 min canter, 1 min walk incl. change of direction, 1 min trot, 1 min canter, 10 s walk). Saliva samples were collected at 0 and 5 min after the test, in accordance with Christensen et al. [12] where saliva cortisol peaked at these times (see also Section 2.2). The lunge test was followed by a gait symmetry test (see Section 2.3) where the horse trotted on a straight line, by hand (6 × 40 m). A fourth saliva sample was taken immediately after the gait symmetry test (Equinosis: EQ), and the horse was walked by hand until collection of the final saliva sample (30 min after completion of the lunge test). After these tests, the horse was measured (height, loin width, chest and cannon bone circumference) and its body condition score was determined using the Henneke BCS 1–9 scale [11]. Weighing of horses was done by loading it into a horse trailer, placed on weight cells (capacity: 6000 kg, precision 0.5 kg, Kern & Sohn, Balingen, Germany), and riders were weighed both without and with equipment (saddle, bridle, saddle mat, boots, helmet, etc.) on a standard bathroom scale (capacity: 150 kg, Beurer, BG13). Finally, rider mobility and balance tests were conducted on the first test day (Table 2; see [13] for further description).

On Test Days 2–4, each rider rode their horse in a standard dressage test in a balanced, crossover design (Figure 1). The riders were fitted with a weight vest, where 1 kg lead blocks could be added to small pockets. The weight was distributed in a balanced way on the rider and a 10 cm wide and 2 m long elastic belt was tightened around the vest to ensure that the vest was firmly attached to the rider and did not move during riding. The treatment order was drawn at random for each rider, while ensuring that all six possible orders were used in each block. The riders were instructed to use the same equipment and ride their horse in the same way on all days. Nosebands were fitted according to national rules, using the official taper gauge from the Danish Riding Organization to ensure a 1.5 cm gap between the nasal bone and the noseband. Saddle and bridle fit were checked manually to ensure that responses would not be caused by ill-fitting equipment. After the warm-up, the rider performed the 5:20 min dressage test, sitting in all gaits. The rider then dismounted and the horse was prepared for the next part, where saddle pressure and gait symmetry were measured (for saddle pressure results, see [13]). These measures were taken separately due to a pilot study where some horses reacted nervously towards the pressure mat and gait symmetry sensors; hence any influence on HR/HRV and cortisol responses to the dressage test was avoided. The rider then re-mounted and rode the horse in two 20 m circles in sitting trot on each rein and finally six straight 40 m lines along the midline of the arena to measure gait symmetry.

### 2.1. Behavior and Heart Rate

The riding tests were recorded on video for later behavioral analysis (Table 3). The riders rode with the empty weight vest in the control treatment to hide the treatment to the video observer. Heart rate (R-R-recordings) was recorded with Polar Equine RS800 CX, consisting of two electrodes, a transmitter and a wristwatch receiver. Water and gel were used to enhance conduction and the electrodes were fitted under the saddle and girth while the transmitter and watch were attached to the saddle. Data were downloaded from the receiver to a computer, using the software Polar ProTrainer 5, Equine Edition. Artefacts in the data were rare, but if they occurred (i.e., apparent as high spikes in the data graph), they were corrected using the error correction function in the software program. A few files were lost due to the rider accidently pushing the stop button during the test ride or an electrode falling off (two in the +25% and one in the +15% treatment). Average and maximum HR (presented as beats per minute (bpm)) were determined for each horse for each test. For the analysis of heart rate variability (HRV), the most informative time and frequency domain parameters were used [14]: RMSSD (ms; root mean square of successive differences between normal heart beats) and LF/HF ratio (%; power of low frequency bands (LF) divided by power of high frequency bands (HF)).

### 2.2. Salivary Cortisol

Saliva was collected with Salivette^®^ (Sarstedt, Nümbrecht, Germany) cotton rolls placed loosely onto the tongue of the horse with forceps for 1 min or until the swab was well soaked. The samples were immediately frozen at −18 °C and were later defrosted and centrifuged for 10 min at 1000× *g*. Concentrations of cortisol were determined using a direct enzyme immunoassay without extraction [15] validated for equine saliva [16]. In this method, the antiserum cross-reacts with cortisol and some cortisol metabolites, and values have to be interpreted as cortisol immunoreactivity. The intra-assay coefficient of variation was 5.0%, the inter-assay variation was 6.7% and the minimal detectable concentration was 0.3 pg/well. All samples were analyzed at the Institute of Animal Science, Aarhus University, Denmark. A total of five samples were lost (4 in the lunge test and one in a +15% riding test, at 5 min).

### 2.3. Gait Symmetry

Gait symmetry was measured and analyzed using the Equinosis Q Lameness Locator with sensors placed on the right forelimb pastern, head and between the tubera sacrale on pelvis. For each horse, vertical head and pelvic movement were measured by uniaxial accelerometers. Differences between the two vertical maximum and minimum head (HDmax, HDmin) and pelvic (PDmax, PDmin) heights between left and right forelimb and hindlimb in trot on a straight line were analyzed as described in [17]. Outliers of the head movement were automatically removed in the software package. The thresholds for asymmetry and corresponding categories are recommendations for clinical use of the system [17]. For the forelimbs, symmetry thresholds values (HDmin, HDmax) were ±6 mm and for hind limbs (PDmin, PDmax) ±3 mm. The resulting categories had thresholds for front limb/hind limb in mm as follows: No asymmetry: 0–6/0–3, mild asymmetry: 6–12/3–6, mild-moderate: 12–18/6–9, moderate: 18–24/9–12, moderate-severe: 24–30/12–15, and severe asymmetry: >30/>15. The levels of evidence of the categories were presented from the software as ‘weak evidence’ (trials with SD > 120% of asymmetry mean); ‘moderate evidence’ (trials with SD > 50% and < 120% of asymmetry mean) and ‘strong evidence’ (trials with SD < 50% of asymmetry mean). For the statistical analysis, recordings of ‘no asymmetry’ and ‘weak evidence of asymmetry’ were categorized as 0 (9% of the recordings were presented as weak evidence). Moderate to strong evidence of mild asymmetry was scored as 1, mild-moderate and moderate asymmetry as 2, and moderate-severe and severe asymmetry was scored as 3. Based on these data, we calculated the sum of the asymmetry scores (total asymmetry score) and the number of affected legs (0–4) for each horse in each treatment.

### 2.4. Data Analysis

The response variables (cortisol concentration; HR (mean), HRV (RMSSD, LF/HF ratio), frequency of conflict behavior (head, mouth, body, tail) and gait symmetry (number of lame legs, total asymmetry score)) were analyzed for an effect of treatment (control (0%), +15% and +25%) in a one-way repeated measures ANOVA (RM ANOVA, SigmaPlot14). A two-way ANOVA was used when appropriate, e.g., to investigate the effect of sampling time and treatment on cortisol responses. Normality of the data was assessed using the Shapiro–Wilk test and variance homogeneity using the Brown–Forsythe test. If data did not meet the assumptions for the model, the Friedman RM ANOVA on ranks was used. In case of significance, the post-hoc tests Bonferroni t-test or Tukey test were used for pairwise multiple comparisons. Data are presented as mean ± se or median [25;75% quartiles]. Data are available as Supplementary Material (Table S1).

### 2.5. Animal Welfare and Ethics

This study was conducted in accordance with national legislation on animal experimentation by the Danish Ministry of Justice, Act. no. 253 (8 March 2013) and § 12 in Act. no. 1459 (17 December 2013), and complies with the EU Directive 2010/63/EU on the use of experimental animals. The study meets the guidelines for ethical treatment of animals in applied animal behavior research by the International Society for Applied Ethology. Riders and horse owners (parents on behalf of minors) gave written consent for their own and their animals’ inclusion in the study.

## 3. Results

**Figure 2:**
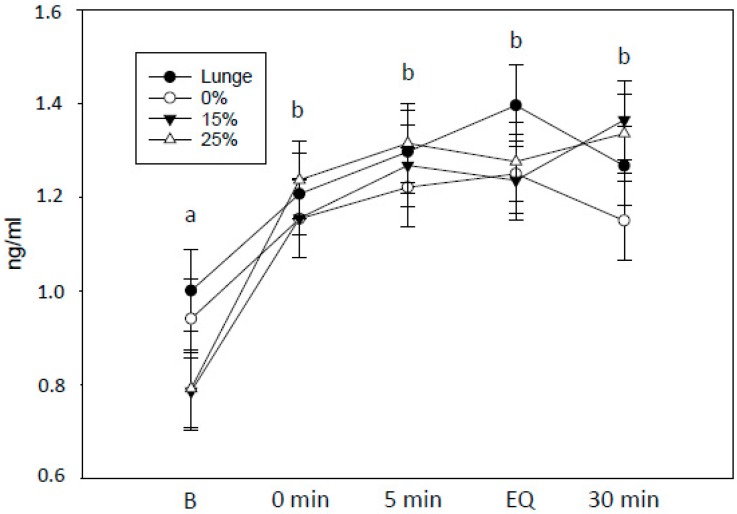
Salivary cortisol concentrations (ng/mL) from the 20 horses (mean ± se) in each treatment, incl. the baseline lunge test. The amount of exercise (warm-up and test) was the same in all treatments. 0% (control): The rider rode without additional weight; 15% and 25%: The rider rode with additional 15% or 25% of her bodyweight, respectively, in a balanced order across three test days. Five saliva samples were collected from each horse per day: B: Baseline, 0 min: Just after the lunge/dressage test, 5 min: 5 min after the lunge/dressage test, EQ: Just after the saddle pressure/gait symmetry test, 30 min: 30 min after the lunge/dressage test. Due to technical issues with the equipment, the duration of the gait symmetry test varied and the EQ and 30 min samples are less informative. Different letters indicate a significant effect of sampling time.

As expected, salivary cortisol concentrations increased in response to exercise, regardless of treatment (two-way RM ANOVA, F_4_ = 10.2, *p* < 0.001; Figure 2), whereas there was no effect of treatment. Due to problems with interference between the lameness detector and the saddle pressure measurements, the duration of the gait symmetry test varied. Thus, the two last saliva samples are less informative (EQ and 30 min).

**Figure 3:**
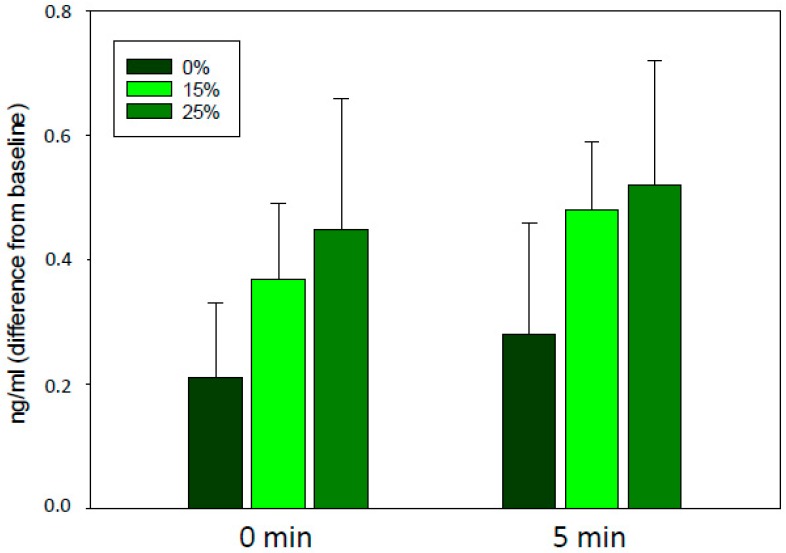
Salivary cortisol concentrations of the 20 horses (ng/mL, mean ± se) calculated as the difference from baseline. 0% (control): The rider rode without additional weight; 15% and 25%: The rider rode with additional 15% or 25% of her bodyweight, respectively, in a balanced order across three test days. 0 and 5 min refer to the sampling time after the dressage test (0 min: F_2_ = 0.58, *p* = 0.56, 5 min: F_2_ = 0.66, *p* = 0.52).

Differences in baseline concentrations between horses further necessitates an analysis of the responses as a difference from baseline values. Although salivary cortisol concentrations appeared to be slightly higher in the +15% and +25% treatment, there was a large individual variation and the difference was not significant (Figure 3).

**Figure 4:**
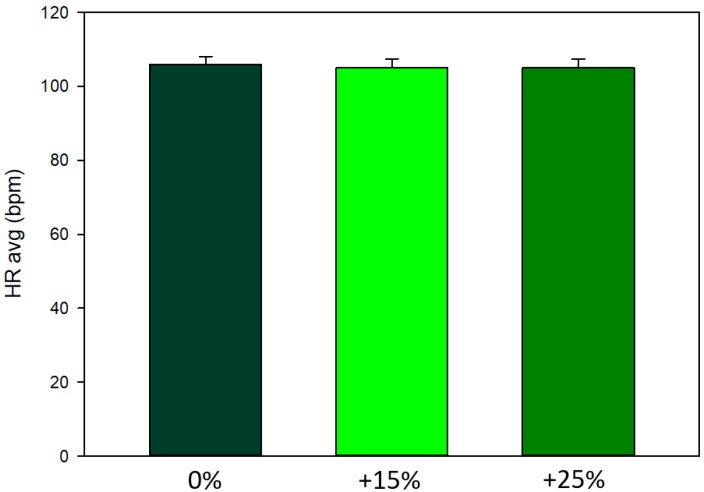
Average heart rates (mean ± se) of the 20 horses did not differ when ridden in a standard dressage test by their usual rider with no additional weight (0%: control), and with additional 15% and 25% of the rider’s bodyweight, in a balanced order across three test days.

There were no overall treatment effects on HR (F_2_ = 0.34, *p* = 0.72; Figure 4), or HRV variables (LF/HF and RMSSD; *p* = 0.70 and *p* = 0.72, respectively). It could be hypothesized that the initial rider:horse bodyweight ratio would affect heart rate responses to the increase in rider weight, i.e., horses with an initially light rider would not respond to the +15 and +25% increase in rider weight but horses with an initially heavier rider would show a response. Thus, to investigate the potential effect of the initial ratio, we added three weight ratio groups based on the 25 and 75% quartiles; Group 1: Initial rider:horse bodyweight ratio <13.8%, Group 2: 13.8–17.1%, Group 3: >17.1%. Surprisingly, horses in the lower rider:horse ratio group (<13.8%) had higher heart rates (two-way RM ANOVA, mean ± se, bpm: Group 1: 116 ± 3.4, Group 2: 102 ± 2.4, Group 3: 104 ± 3.4, F_2_ = 6.25, *p* = 0.009, and no interaction). However, the horses in Group 1 also appeared to have somewhat higher heart rates in the baseline lunge test (one-way ANOVA, Weight Group 1: 114 ± 1.8, Group 2: 107 ± 3.3, Group 3: 105 ± 6.5, *p* = 0.35), suggesting that other factors such as baseline fitness may be involved.

The occurrence of conflict behavior varied largely between horses. Five horses never showed tail swishing during the three riding tests, whereas one horse showed 155 (control), 184 (+15%) and 137 (+25%) tail swishes. Tail swishing was the most frequently recorded behavior (total 1196 recordings, shown by 15 horses), followed by open mouth (total 227 recordings, shown by 8 horses) and head movements (total 106 recordings, shown by 5 horses). The occurrence of the different types of conflict behavior did not correlate, e.g., horses that showed tail swishing were not more likely to show mouth or head movements (Spearman, all *p* > 0.05). Due to the many zeros in the data set, we created a joint variable with all the recorded behaviors (Table 3; conflict behavior, shown by 19 of the 20 horses) but found no effect of weight treatment (Friedman RM ANOVA on ranks, median [25,75%]: control: 9.0 [3,27], +15%: 8.5 [1,29], +25%: 8.0 [2,35], *p* = 0.61).

The gait symmetry measures (number of affected legs, total asymmetry score) were unaffected by the weight increase, as well as whether the horse was ridden or not (one-way RM ANOVA; number of affected legs/horse: lunge: 1.20 ±0.20; control (0%): 1.20 ± 0.19; +15%: 0.90 ± 0.18; +25%: 0.85 ± 0.13, F_3_ = 1.16, *p* = 0.33; and total asymmetry score: lunge: 1.40 ± 0.22; control (0%): 1.80 ± 0.36; +15%: 1.15 ± 0.29; +25%: 1.05 ± 0.22, F_3_ = 1.42, *p* = 0.24). There was also no effect of weight ratio class (1–3).

## 4. Discussion

This study found no effect on behavioral, physiological and two gait symmetry parameters in horses exposed to an acute increase in rider weight by 15% and 25% of the regular rider’s bodyweight. Interestingly, the rider:horse bodyweight ratios in our study overlap with the ratios in Dyson et al. [5] where the heavy rider was 15.3–17.9% of the horse’s bodyweight. In that study, all riding tests with the heavy rider were abandoned on welfare grounds, based on a subjective scoring of lameness and behavioral markers of pain. The result was not replicated in our study, although the rider:horse bodyweight ratio was 15–23% in the +25% treatment. The duration of exercise (including warm-up, riding test and gait symmetry testing) appear to be similar in the two studies. However, we could not detect changes in gait symmetry in this study, which may relate to differences in the seat position of the riders, as the riders in the present study were sitting compared to rising trot in the study by Dyson et al. [5]. Rising trot is known to induce movement asymmetries in the horse that may not be related to pain [18]. Our study was a crossover study where each rider rode her own horse in each treatment, whereas four riders of different weight comprised the treatment in Dyson et al. [5]. Rider skill and balance as well as other factors, such as individual saddle fit, are likely to influence conflict behavior and weight bearing capacity in horses, and isolating the effect of rider weight while keeping other factors identical is necessary to gain knowledge of the effect of increased rider weight per se. In our study, the large individual variation in cortisol concentrations and especially in conflict behavior likely reflects individual rider style [12]. A larger study with a number of riders of the same weight, riding the same horses in a standardized test would enable an analysis of the effect of rider style.

Our results add to the existing literature where higher rider:horse bodyweights have been investigated and often at higher exercise intensities e.g., [2,3,4] compared to the low exercise intensity in this study. For example, heart rates reached 187 bpm (20%) and 199 bpm (35%) in the study on Icelandic horses by Stefánsdóttir et al. [2]. In comparison, the average HR across treatments was 105 bpm in this study on dressage horses. In a previous study, we measured HR of dressage horses in a riding test with different head-and-neck positions. In that study, the average HR was 116 in a 10-min dressage test consisting of 5 min trot, 4 min canter and 1 min walk [12]. However, the participating dressage riders remarked that the test was too long and they would not normally ride 9 min in trot and canter without a short break in walk, and we therefore decreased the exercise intensity in the present study. We aimed to mirror the daily training situation that many leisure horses are exposed to, in order to evaluate whether relatively small increases in rider weight influences acute responses in horses also at lower exercise intensities, as initially suggested by Dyson et al. [5]. Acute changes in gait symmetry may be a sign of orthopedic pain, caused by, e.g., overloading of anatomical structures or subclinical problems. Long-term effects of gait asymmetry, resulting in uneven loading of the limbs, can potentially lead to health issues [5,18]. Similarly, increased levels of conflict behavior can be indicative of discomfort or pain with negative impact on horse welfare, health and performance. Physiological responses, reflecting increased activity of the autonomic nervous system (HR/HRV) or the Hypothalamic-Pituitary-Adrenal (HPA)-axis (cortisol) are common when the physical workload increases, e.g., when the weight load increases, and may decrease as the individual adapts, unless related to other psychological or physical stressors. Thus, these parameters should be interpreted in combination with behavioral and gait symmetry data. The surprisingly similar heart rates of the horses in the different treatments in the present study suggest that the increased rider weight did not impose a significantly higher physical workload for the horses. The cortisol data may suggest an increase in response to higher weight loads but due to the high individual variation, further studies are needed to verify this. Previous experiments have found saliva cortisol to be more sensitive than HR/HRV data [12,19], and a new study even cautions against the use of HRV data measured with Polar monitors on horses due to lack of validation [20].

Given the low exercise intensity in the present study, one should not extrapolate the results to other exercise intensities and disciplines. It is possible that an acute increase in rider weight, even within the range of rider:horse bodyweight ratios applied in the present study, can lead to behavioral, physiological and gait parameter changes at higher exercise intensities and in other disciplines, e.g., show jumping. Additionally, there may be long-term effects of an increased weight load, and further studies are required to study long-term effects on horse health and performance.

## 5. Conclusions

Increasing rider weight by 15% and 25% did not result in significant short-term alterations in heart rate, salivary cortisol, behavior and gait symmetry in dressage horses during a standard dressage test. Maximum horse:rider weight ratios were 15–23%. Thus, within this weight ratio range and during light exercise, acute increases in rider weight did not induce changes in these parameters. Further studies on the effect of rider weight in various equestrian disciplines as well as long-term effects are required before appropriate guidelines for rider weight can be developed.

## Figures and Tables

**Table 1:** Conformation and weight of the horses/ponies and their riders.

Parameter	Horses	Ponies
Number	17	3
Height (cm), mean ± se (range)	168 ± 1.1 (160–177)	144 ± 4.2 (136–148)
Weight (kg)	577 ± 11.5 (500–650)	349 ± 29.4 (290–380)
Body Condition Score ^1^	5.8 ± 0.8 (5–7.5)	5.7 ± 0.3 (5–6)
Chest circumference (cm)	198 ± 1.7 (182–210)	166 ±5.4 (155–172)
Age (yrs)	8.4 ± 0.5 (5–14)	14.7 ± 3.2 (11–21)
Cannon bone circumference (cm)	21.6 ± 0.2 (21–23)	18 ± 0.8 (17–19)
Loin width (cm)	55.6 ± 0.9 (50–63)	45.3 ± 1.9 (43–49)
Rider weight (kg)	70.4 ± 2.1 (55–83)	49.7 ± 5.3 (39–56)
Rider weight incl. tack (kg)	85.5 ± 2.3 (68–100)	63.4 ± 5.8 (52–71)
Rider age (years)	35.9 ± 2.5 (20–54)	12.0 ± 0.6 (11–13)
Rider:Horse bodyweight ratio (%) ^2^	14.8 ± 0.37 (12.4–18.1)	18.2 ± 0.40 (17.6–18.9)

^1^ Henneke Body Condition Score, scale 1–9 [11]; ^2^ Rider weight includes tack.

**Table 2:** Overview of Tests and Test Days.

Day	1	2	3	4
Activities	Clinical examination Baseline lunging test Baseline gait symmetry test Weighing of horse and rider Horse conformation Rider mobility and balance tests (on-ground)	Dressage and gait symmetry; Test 1: control (0%), +15% or +25%	Dressage and gait symmetry; Test 2: control (0%), +15% or +25%	Dressage and gait symmetry; Test 3: control (0%), +15% or +25%

**Table 3:** Ethogram of recorded behavior.

Behavioral Category (Freq)	Description
Gait change	The horse makes an undesired gait change, e.g., from canter to trot during the 1 min canter sequence, or from walk to trot during the 1 min walk sequence
Other undesired body movements	Bucking, kicking
Head movements	Head tossing, shaking or raising (i.e., movements of the head away from the usual head-and-neck position)
Tail movements	Lateral, dorsoventral or circular motion of the tail that interrupts the rhythmical waving motion of the tail corresponding to the gait
Open mouth	Mouth clearly opened; lower or upper teeth are visible

## Supplementary Materials

The following are available online at https://www.mdpi.com/2076-2615/10/1/95/s1, Table S1: Data table.
